# Influence of Personality, Resilience and Life Conditions on Depression and Anxiety in 104 Patients Having Survived Acute Autoimmune Thrombotic Thrombocytopenic Purpura

**DOI:** 10.3390/jcm10020365

**Published:** 2021-01-19

**Authors:** Tanja Falter, Sibylle Böschen, Markus Schepers, Manfred Beutel, Karl Lackner, Inge Scharrer, Bernhard Lämmle

**Affiliations:** 1 Institute of Clinical Chemistry and Laboratory Medicine, University Medical Center of the Johannes Gutenberg University, 55131 Mainz, Germany; Sibylle.boeschen@web.de (S.B.); Karl.Lackner@unimedizin-mainz.de (K.L.); 2 Institute of Medical Biostatistics, Epidemiology and Informatics (IMBEI), University Medical Center of the Johannes Gutenberg University, 55131 Mainz, Germany; Markus.Schepers@uni-mainz.de; 3 Department of Psychosomatic Medicine and Psychotherapy, University Medical Center of the Johannes Gutenberg University, 55131 Mainz, Germany; Manfred.Beutel@unimedizin-mainz.de; 4 Center for Thrombosis and Hemostasis (CTH), University Medical Center of the Johannes Gutenberg University, 55131 Mainz, Germany; Inge.scharrer@unimedizin-mainz.de (I.S.); Bernhard.laemmle@uni-mainz.de (B.L.); 5 Department of Hematology and Central Hematology Laboratory, Inselspital, Bern University Hospital, University of Bern, CH 3010 Bern, Switzerland; 6 Haemostasis Research Unit, University College London, London WC1E 6BT, UK

**Keywords:** thrombotic thrombocytopenic purpura, depression, resilience, quality of life

## Abstract

Autoimmune thrombotic thrombocytopenic purpura (iTTP) is a life-threatening, relapsing disease in which an acquired deficiency of the enzyme ADAMTS13 leads to generalised microvascular thrombosis. Survivors have a high prevalence of depression and impaired cognitive function. The aim of this study was to determine whether life circumstances and personality have an influence on the development and severity of depression and anxiety in iTTP patients and how they impact the quality of life. With validated questionnaires, we examined the prevalence of depression and anxiety symptoms in 104 iTTP patients, as well as parameters of subjective cognitive deficits, quality of life, attitude to life and resilience. iTTP patients had significantly more depressive symptoms (*p* < 0.001), a tendency to have anxiety disorders (*p* = 0.035) and a significantly worse cognitive performance (*p* = 0.008) compared to the controls. Sex, age, physical activity and partnership status had no significant influence on depression, whereas the number of comorbidities did. Lower scores of resilience, attitude to life and quality of life were reported by patients compared to controls. iTTP patients had a high prevalence of depression and anxiety, as well as a more negative attitude to life and low resilience. Resilience correlated negatively with the severity of the depression. Furthermore, quality of life and cognitive performance were significantly reduced.

## 1. Introduction

Autoimmune thrombotic thrombocytopenic purpura (iTTP) is a potentially life-threatening, relapsing disease in which an acquired deficiency of the von Willebrand factor (VWF)-cleaving protease, ADAMTS13, leads to generalised microvascular thrombosis in various organs [1]. The characteristic features are thrombocytopenia due to the consumption of platelets and microangiopathic haemolytic anaemia with destruction of erythrocytes [2]. As soon as the laboratory parameters return to normal after treatment of an acute bout, the patient is often regarded as cured but lives with the risk of suffering an acute relapse at any time [3]. In recent years, some studies have shown that iTTP is much more than just an acute disease; not only potential relapses but also long-term consequences of the past acute episode should be in focus. Besides neurological impairments [4,5,6,7], depression is a prevalent sequela [6,7,8,9,10]. The occurrence of depression and anxiety disorders has been documented in numerous other acute and chronic diseases, e.g., stroke, multiple sclerosis and cancer [11,12,13,14,15]. Depression itself is considered a risk factor for cardiovascular disease [16] and leads to increased morbidity and mortality, regardless of its severity [15,17]. Furthermore, depression causes a reduced quality of life for patients and lower resilience. In turn, individuals with low resilience are more prone to develop psychiatric disorders [18]. However, resilience is also significantly influenced by other factors, such as alexithymia [19].

We [10], as well as others [6,9,20], have shown that the prevalence of depression is significantly increased in patients that have survived acute iTTP. In addition, our results revealed that the severity of the acute iTTP episode is not the determining factor for the development and severity of depression [10].

The aim of the present study was to determine whether life circumstances (e.g., partnership, employment and physical activity), personality and resilience are associated with the development and severity of depression and anxiety in iTTP patients and how they influence their quality of life.

## 2. Materials and Methods

The results are part of a five-year prospective cohort study that was approved by German law (Landeskrankenhausgesetz §36 and §37) in accordance with the Declaration of Helsinki and by the local Ethics Committee of “Landesärztekammer Rheinland-Pfalz” (837.265.14 (9504-F)), where all participants gave written consent to participate.

The study was divided into two main themes. The first part referred to evaluations in 2013 and 2014 that have already been published [10]. In brief, the iTTP patients displayed a high prevalence of depression and cognitive deficits via self-reporting questionnaires. However, we did not detect a significant correlation between the severity of depression or cognitive deficits and the number or severity of acute TTP episodes. Nevertheless, we could demonstrate a highly significant correlation between the severity of depression and the degree to which cognitive performance was reduced [10].

The second part had a focus on the long-term psychological consequences, where the personality structure and the influence on the quality of life were examined in more detail here (Figure S1).

In 2015 and 2016, using validated questionnaires, we examined the prevalence of depressive (PHQ-9) and anxiety symptoms (GAD-7) in 104 iTTP patients, as well as parameters of subjective cognitive deficits (FLei), resilience (RS-11), attitude to life (LOT-R) and quality of life (QLQ-C30) at two observation points one year apart. At the second observation time, an age- and sex-matched healthy control group was simultaneously interviewed.

### 2.1. Patients and Controls

The patient cohort for this study was recruited from the iTTP patients that were treated directly at the University Hospital Mainz, as well as from external patients for whom the University Hospital Mainz was asked for medical advice by external clinics. The external patients that presented themselves personally at the University Hospital Mainz at least once were asked to participate in the study. Since October 2012, all patients over 18 years of age with a confirmed iTTP diagnosis (defined as microangiopathic haemolytic anaemia, thrombocytopenia (<150,000/µL), severe acquired ADAMTS13 deficiency (activity < 10%) and an ADAMTS13 inhibitor (>0.5 Bethesda units/mL)) in the acute TTP episode have been included.

The healthy controls were 300 randomly selected people that were age- and gender-matched to the iTTP collective, whose contact details were received from the residents’ registration office. We received 134 evaluable questionnaires.

### 2.2. Psychometric Assessment

TTP patients were invited to participate in the study twice with an interval of 10 to 12 months. At both time points, psychometric questionnaires were either sent by regular mail to the patients’ home or directly given to patients when they presented at the outpatients ward.

One patient was excluded from this study (in 2015/2016) because of an inability to answer the questionnaires after having suffered from ischemic brain damage during an acute TTP episode.

#### 2.2.1. Patient Health Questionnaire 9 Items (PHQ-9)

The Patient Health Questionnaire 9 (PHQ-9) was developed in 2001 by Spitzer et al. and is indicated for the self-assessment of depressive symptoms and their classification into degrees of severity [21]. It consists of nine questions, each of which is attributed 0, 1, 2 or 3 points. The final score is calculated from the sum of all answers. A high score indicates that patients often show depressive symptoms. If the patient receives 0 to 4 points, it can be assumed that there is no depression. Mild depressive symptoms are present at 5 to 9 points, moderate symptoms at 10 to 14 points and moderate-to-severe depression at 15 to 19 points. A score ≥ 20 points signals severe depressive symptomatology. The presence of major depression can be assumed at a cut-off of ≥10 points.

#### 2.2.2. Generalized Anxiety Disorder 7 (GAD-7)

The “Generalized Anxiety Disorder 7”, which is a self-assessment questionnaire with seven items, was developed to diagnose and classify generalised anxiety disorders [22]. The GAD-7 examines the symptoms of anxiety, such as nervousness or irritability in seven items. The patient must evaluate how often these symptoms have been experienced in the last 2 weeks. Depending on the answer, the patient receives between 0 and 3 points. The sum of all seven items corresponds to the total score. If the total score is between 0 and 4 points, no anxiety disorder can be assumed. A score of 5 points or more indicates a mild anxiety disorder, 10 points or more indicates a moderate anxiety disorder and 15 points or more indicates a severe anxiety disorder.

#### 2.2.3. FLei

Cognitive deficits were assessed using the German questionnaire for complaints of cognitive disturbances (FLei), which is a self-report measure with 30 items covering the domains of deficient attention, memory and executive functions, with 10 items each. All items are rated on a five-point Likert-scale (0 = at no time; 4 = very frequent). Accordingly, the total score for all 30 items ranges between 0 and 120 points. The internal consistencies of the three subscores (Cronbach’s alpha and split-half reliability) are all >0.87 [23]. Healthy controls reported in the literature showed a mean of 29.1 (SD 18.7), whereas controls with major depression (ICD.10) had a mean of 56.5 (SD 23.1) [23].

#### 2.2.4. Resilience Scale 11 (RS-11)

The Resilience Scale 11 (RS-11) was developed as a tool to measure the mental resistance of patients [24]. The self-assessment questionnaire consists of 11 questions, each of which is rated with 1 to 7 points. From these scales, an overall score is formed, with values from 11 to 77. The higher the total score, the higher the presumed resilience of the respondent.

#### 2.2.5. Life Orientation Test–Revised (LOT-R)

The Life Orientation Test–Revised (LOT-R) is a questionnaire with 10 items, each with five possible answers, which serves to assess the attitude to life. It evaluates general character features, such as the tendency toward optimism and pessimism, for both of which, a subscore is given. In addition, an overall score can be calculated [25].

#### 2.2.6. Quality of Life Questionnaire C 30 (QLQ-C30)

The Quality of Life Questionnaire C 30 (QLQ-C30) was developed in 1993 by the European Organisation for Research and Treatment of Cancer to specifically evaluate the quality of life of cancer patients [26,27]. Fifteen subscales are formed from the 30 items. The subscales consist of five function scales (physical, role, cognitive, emotional and social function), three symptom scales (fatigue, pain and nausea or vomiting) and a global health status/quality of life scale, as well as six individual items with specific symptoms (dyspnea, loss of appetite, insomnia, constipation and diarrhea, and a question on the financial impact of the disease). Each item has four response alternatives, except for the global health status/quality of life scale, which has response options ranging from 1 to 7.

### 2.3. Covariates

In addition to the questionnaires, personal information, such as age, gender and life circumstances such as partnership and number of children, were also collected. Furthermore, data on physical fitness and other chronic and acute illnesses were obtained. The participants were able to specify their physical fitness themselves with the help of five predefined answer options (from not at all or only a little bit (1–2 times per month) to extremely active (more than 5 times per week)). Fifteen comorbidities were specifically asked for and further comorbidities could be indicated.

### 2.4. Statistical Analyses

Statistical analyses were performed using SPSS version 22.0 (IBM GmbH, Ehningen, Germany). Missing data were imputed using median imputation. The descriptive statistics included frequency, mean, standard deviation, median, interquartile range (IQR), minimum and maximum. The differences between the two groups were tested using Student´s *t*-test for normally distributed data and the non-parametric Mann–Whitney *U* test for non-normally distributed data. For comparing changes in the different scores of patients who completed both surveys (in 2015 and 2016), a dependent *t*-test was used, as well as the dependent Wilcoxon test. Spearman’s rank correlation coefficient (*r_s_*) was calculated to estimate the relationship between depressive symptoms and resilience, respectively. The correlations between age, gender, comorbidities, physical activity and depressive symptoms were determined using Pearson’s correlation coefficient (*r*). Any *p*-values less than 0.05 were considered to be statistically significant.

## 3. Results

### 3.1. Study Population

**Figure 1:**
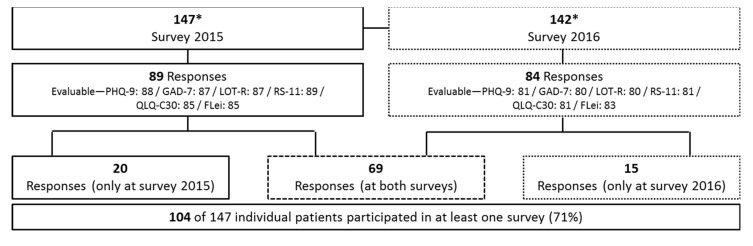
Patient recruitment and response rates in two surveys of the cohort of autoimmune thrombotic thrombocytopenic purpura (iTTP) patients from Mainz. A total of 147 eligible iTTP patients in remission were invited to fill in the various questionnaires used in two surveys each (2015 and 2016). * Between the first and second survey five patients were lost to follow-up. Questionnaires concerned: depression (PHQ-9), anxiety (GAD-7), attitude of life (LOT-R), resilience (RS-11), quality of life (QLQ-C30) and cognitive disturbance (FLei).

From June 2015 until July 2016, 147 patients with an acquired TTP that was diagnosed prior to starting this study were asked to participate. Between the 2015 and 2016 surveys, five of the 147 participants were lost to follow-up. Accordingly, 142 TTP patients were sent the questionnaires in the 2016 survey, about one year after the first inquiry. We received 89 responses in the 2015 survey, with 89 of those being evaluable for RS-11, 88 for PHQ-9, 87 for GAD-7 and LOT-R and 85 for FLei (Figure 1). Eighty-four responses were obtained in the 2016 survey, with 83 of those being evaluable for Flei, 81 for PHQ-9, RS-11 and QLQ-C30 and 80 for GAD-7 and LOT-R (Figure 1).

Overall, we received responses from 104 individual iTTP patients, 69 answered both surveys, 20 participated only in 2015 and 15 only in 2016 (Figure 1). The depression, anxiety, impairment of cognitive performance, resilience, attitude of life and quality of life results of the iTTP patients were compared with those from 134 healthy controls.

### 3.2. Patient Characteristics

A total of 147 (2015) and 142 (2016) iTTP patients could be reached for the surveys. The response rate was 60% in the 2015 survey and 59% in the 2016 survey (Figure 1). The characteristics of the patients and the healthy controls are shown in Table 1.

About 80% of the patients were female and the median ages were 48 and 51 years in 2015 and 2016, respectively. Half of the participating patients were employed and one third were retired. The majority of patients lived in a partnership and 63% and 65% in 2015 and 2016, respectively, had children. One-third of patients each took part in low, intermediate or high physical activity. The median body mass index (BMI) was 26 and 28 kg/m^2^, respectively, in the two surveys, with 27% and 35% being obese. Overall, the iTTP collective had several other diseases besides iTTP (78% had comorbidities in 2015 and 88% in 2016). On average, the iTTP patients had one additional disease in 2015 (min 0, IQR 1–3, max 9) and two additional comorbidities in 2016 (min 0, IQR 1–3, max 9). The control group had substantially fewer diseases (median 1, min 0, IQR 0–2, max 7). The comorbidities that were explicitly asked for were chronic heart diseases, hypertension, gastrointestinal diseases, rheumatoid arthritis, diabetes mellitus, skin diseases, metabolic disorders, allergies, multiple sclerosis, chronic pulmonary diseases, chronic pain, thyroid diseases, obesity and cancer. In addition, comorbidities not listed could be indicated. Both in iTTP patients and the control group, the most frequent health problems were hypertension and thyroid diseases, followed by allergies. Compared to the control collective, the iTTP patients were significantly more overweight, were more often smokers and had more comorbidities (Table 1).

### 3.3. Depression (PHQ-9)

**Figure 2:**
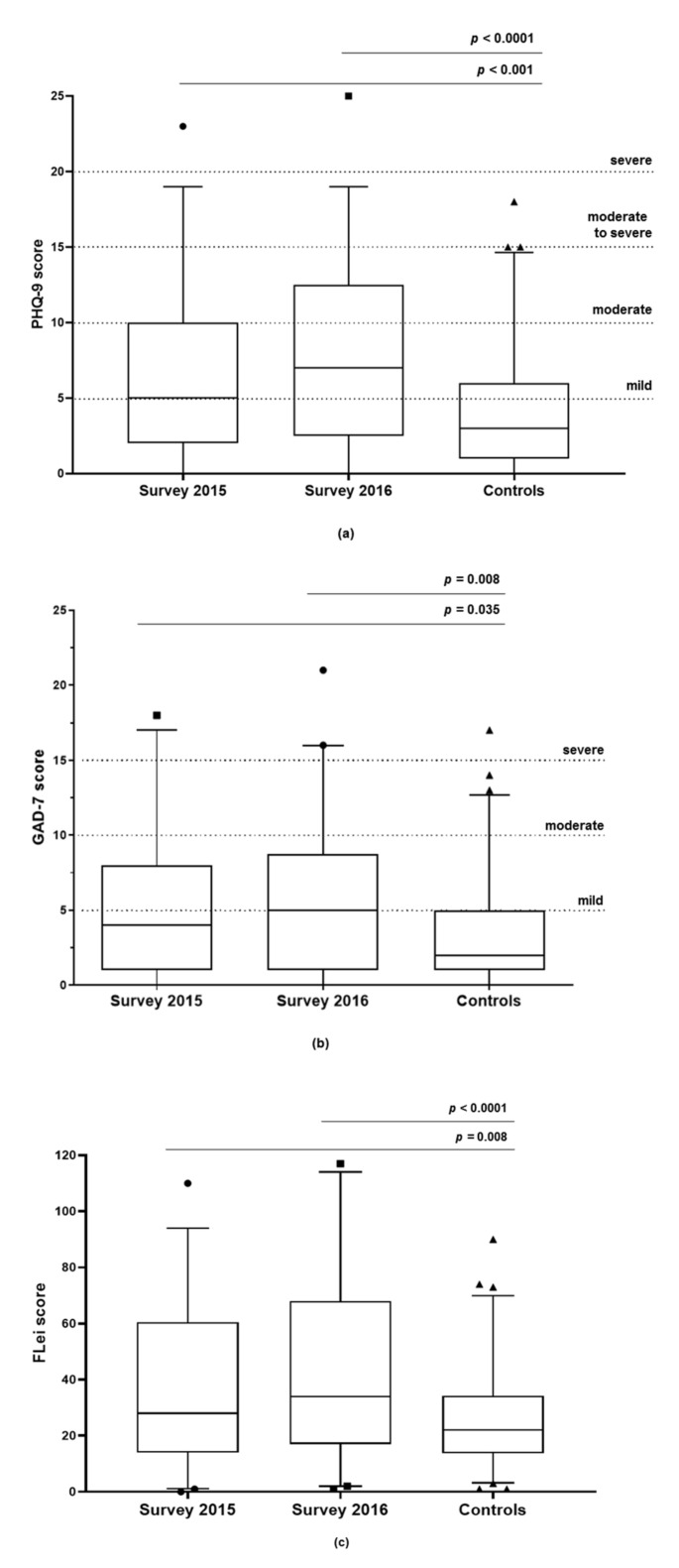
Results of the depression (PHQ-9), anxiety disorder (GAD-7) and cognitive performance (FLei score) questionnaires from the iTTP patients in two surveys (2015 and 2016) and the healthy controls (median, box 25th and 75th percentiles, whiskers 2.5th and 97.5th percentiles, ●, ▪, ▲ denote outliers above 97.5th percentiles or below 2.5th percentiles outliers). (**a**) PHQ-9: For the first survey (*n =* 88), the median evaluated score was 5 (IQR 2–10), for the second survey (*n =* 81), the median score was 7 (IQR 2.5–12.5), and for the healthy controls, the median score was 3 (IQR 1–6). (**b**) GAD-7: For the first survey (*n* = 87), the median evaluated score was 4 (IQR 1–8), for the second survey (*n* = 80), the median score was 5 (IQR 1–8.75), and for the healthy controls (*n* = 131), the median score was 2 (IQR 1–5). (**c**) FLei: For the first survey (*n* = 85), the median evaluated score was 28.0 (IQR 14–60.5), for the second survey (*n* = 81), the median score was 34.0 (IQR 17–68), and for the healthy controls (*n* = 130), the median score was 22.0 (IQR 13.75–34.25).

In 2015, 54 (61.4%) of 88 iTTP patients were scored as having current depressive symptoms by the PHQ-9 (score ≥ 5) and the proportion of patients with major depression (score ≥ 10) was 21.6%. The median score was 5 (IQR 2–10), ranging from 0 to 23 (Figure 2a). Thirty-four (38.6%) patients had no depression, 31 (35.2%) had mild depression, 13 (14.8%) had moderate depression, nine (10.2%) had moderate-to-severe depression and 1 (1.1%) had severe depression.

In 2016, 51 (63.0%) of 81 iTTP patients were scored as having current depressive symptoms by the PHQ-9 (score ≥ 5) and the proportion of patients with major depression (score ≥ 10) was 34.5%. The median total score was 7 (IQR 2.5–12.5), ranging from 0 to 23 points (Figure 2a). Regarding the severity of depression, 30 (37.0%) patients had no depression, 23 (28.4%) had mild depression, 14 (17.3%) had moderate depression, 13 (16.0%) had moderate-to-severe depression and 1 (1.2%) had severe depression.

Forty-five of 133 (33.8%) healthy controls had depressive symptoms as scored by the PHQ-9 (score ≥ 5) (Figure 2a). Six (4.6%) of the 133 controls had clinically relevant depression (score ≥ 10). The median total score was 3 (IQR 1–6), ranging from 0 to 18 points (Figure 2a). The prevalence of depression in iTTP patients was significantly higher in both surveys (2015 *p* < 0.001; 2016 *p* < 0.0001) than in the controls (Figure 2a). No difference in the prevalence or severity of depression in the iTTP patients was found between the two surveys.

### 3.4. Anxiety Disorder (GAD-7)

In 2015, 49 (56.3%) of 87 iTTP patients had no symptoms of anxiety, whereas 21 (24.1%) had mild anxiety (score 5–9), 12 (13.8%) had moderate anxiety (score 10–14) and five (5.7%) had severe anxiety (score 15–21). The median evaluated score was 4 (IQR 1–8), ranging from 0 to 18 (Figure 2b).

In 2016, 37 (46.3%) of 80 iTTP patients had no symptoms of anxiety, whereas 29 (36.3%) patients had mild anxiety (score 5–9), 11 (13.8%) had moderate anxiety (score 10–14) and three (3.8%) had severe anxiety (score 15–21). The median evaluated score was 5 (IQR 1–9), ranging from 0 to 21 (Figure 2b). Ninety-five of the 132 controls (72.0%) did not show any symptoms of anxiety (Figure 2b). The prevalence of anxiety disorders in the overall iTTP cohort was higher in both surveys (2015 *p* < 0.035; 2016 *p* < 0.008) than in the control group (Figure 2b). In particular, the proportion of clinically relevant anxiety disorders (score ≥ 10) in the iTTP cohort was significantly higher in 2015 (19.5%) and in 2016 (17.6%) than in the control group (8.4%).

### 3.5. Cognitive Performance (FLei Score)

Eighty-five iTTP patients in 2015 and 81 in 2016 were evaluable for their cognitive performance using FLei (Figure 2c). The total scores in both surveys were normally distributed and showed a median of 28.0 (IQR 14–60.5) in the 2015 survey and a median of 34.0 (IQR 17–68) in the 2016 survey, ranging from 0 to 117 (Figure 2c). Cognitive performance was significantly worse for iTTP patients in both surveys (*p* = 0.008 for 2015, *p* < 0.0001 for 2016) in comparison to the healthy cohort (median 22.0, IQR 13.75–34.25) (Figure 2c).

### 3.6. Resilience (RS-11)

**Figure 3:**
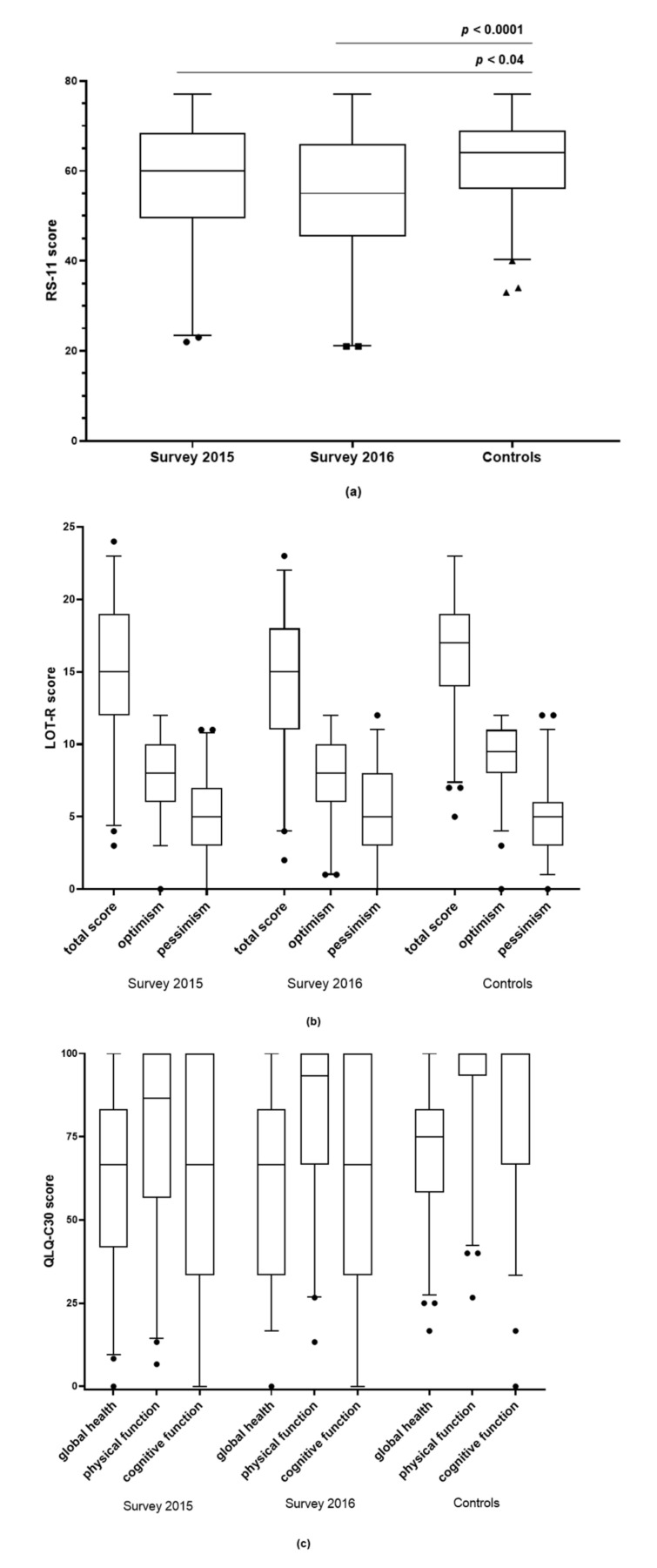
Results of the resilience (RS-11), attitude to life (LOT-R) and quality of life (QLQ-C30) questionnaires from the autoimmune thrombotic thrombocytopenic purpura (iTTP) patients in two surveys (2015 and 2016) and the healthy controls (median, box 25th and 75th percentiles, whiskers 2.5th and 97.5th percentiles, ●, ▪, ▲ outliers above the 97.5th percentiles or below the 2.5th percentiles). (**a**) RS-11: For the survey in 2015, the median evaluated score was 60 (IQR 49.5–68.5), for the survey in 2016, the score was 55 (IQR 45–66), and for healthy controls, the score was 64 (IQR 56–69). (**b**) LOT-R: In the optimism score, the patients showed significantly worse results than the control group in both rounds (2015 survey *p* = 0.011, 2016 survey *p* = 0.006). Within the pessimism score, no large differences between the patients and controls could be detected (2015 survey *p* = 0.49, 2016 survey *p* = 0.63). In the first round, no significant difference (*p* = 0.088) between the patients and controls was found in the total score, but in the second round, a significant difference (*p* = 0.009) between the patients and controls was found. (**c**) QLQ-C30: In the “global health”, “physical function” and “cognitive function” scores, the patients had significantly worse results than the control group in both rounds (2015 and 2016 surveys *p* < 0.0001).

The 89 iTTP patients in the first survey in 2015 showed a median score of 60 (min 22, IQR 49.5–68.5, max 77) and the 81 iTTP patients in the second survey 2016 showed a median score of 55 (min 21, IQR 45–66, max 77) (Figure 3a). The control collective of 129 persons had a median score of 64 (min 33, IQR 56–69, max 77) (Figure 3a). Thus, the survivors of iTTP, both in the first (*p* < 0.04) and second (*p* < 0.0001) surveys, exhibited a lower resilience than the control collective (Figure 3a).

### 3.7. Attitude to Life (LOT-R)

The questionnaire on the attitude to life (LOT-R) was answered by 87 patients in 2015 and 80 patients in 2016. The results in the categories of optimism, pessimism and the total score could be compared with 134 control persons. In the first survey, no significant difference (*p* = 0.088) between the patients (median 15, IQR 12–19) and controls (median 17, IQR 14–19) was found in the total score, but in the second survey, a significant difference (*p* = 0.009) between the patients (median 17, IQR 11–18) and controls was found (Figure 3b). In the optimism score, the patients showed significantly worse results than the control group in both rounds (2015 survey *p* = 0.011, 2016 survey *p* = 0.006) (Figure 3b). Within the pessimism score, no large differences between the patients and controls could be detected (2015 survey *p* = 0.49, 2016 survey *p* = 0.63) (Figure 3b).

### 3.8. Quality of Life (QLQ-C30)

Eighty-five TTP patients in 2015 and 81 patients in 2016 were evaluable regarding their quality of life using the QLQ-C30 (Figure 3c). They could be compared with 134 healthy controls (Figure 3c). In all five functional scales (physical, cognitive, role and social function *p* < 0.0001 for both 2015 and 2016; emotional function *p* = 0.001 for 2015/*p* = 0.007 for 2016), as well as in the global quality of life scale (*p* = 0.001 for 2015/*p* = 0.007 for 2016), the iTTP patients showed significantly worse results in both rounds than the control group (Figure 3c, not all five functional scales are shown).

### 3.9. Correlation of Life Circumstances and Personality with Depression

Sex, age, physical activity and partnership status were not significantly correlated with depression. Using Pearson’s correlation (age, physical activity, partnership status) and Mann–Whitney *U* analysis (sex), no significant correlation was established for any of these parameters in 2015 or 2016 with the degree of depression (PHQ-9 score) (Table S1). The comorbidities were associated with the PHQ-9 score. Only the number of co-morbidities was considered, not the specific diseases. If a patient had more co-morbidities, the PHQ-9 score showed a higher value, i.e., a more severe depressive state (*p* = 0.015 for 2015/*p* = 0.006 for 2016) (Table S1). Furthermore, the correlation between the QLQ-C30 score (quality of life) and the PHQ-9 score was significant (*p* < 0.0001 for both 2015 and 2016) (Table S1).

### 3.10. Correlation of Resilience with Depression

**Figure 4:**
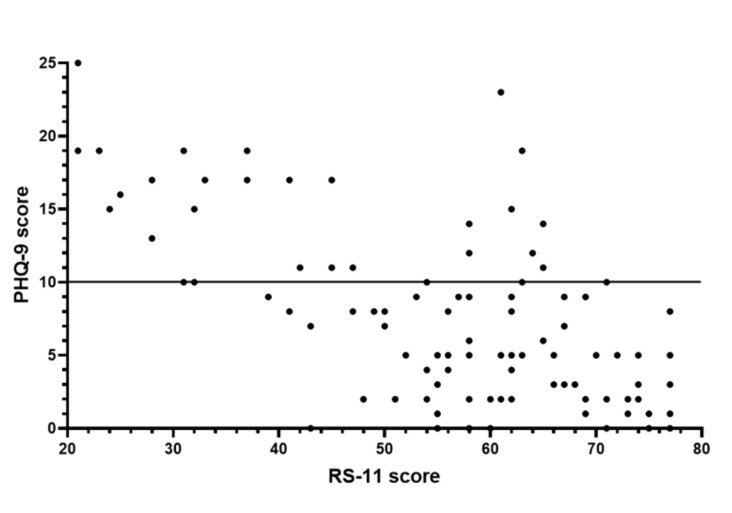
Correlation of the PHQ-9 score (depressive symptoms) with the resilience score. The correlation of the degree of depression (PHQ-9) with resilience (RS-11) was analysed for 102 iTTP patients (*r_s_* = −0.588, *p* < 0.0001) (every iTTP patient was analysed only once, the first evaluation of those that participated in both surveys was considered). The horizontal line indicates the cut-off for major depression (PHQ-9 score ≥10).

Our data revealed that the degree of depression (PHQ-9) was negatively associated with resilience (RS-11). Spearman’s rank correlation coefficient (*r_s_*) for 88 iTTP patients in the 2015 survey was −0.5346 (*p* < 0.0001), and for the 78 participants in the 2016 survey, *r_s_* = −0.6447 (*p* < 0.0001). In Figure 4, the RS-11 and PHQ-9 data for 102 individual iTTP patients (only the first survey was considered for patients who participated in both surveys) revealed an *r_s_* of −0.5878 (*p* < 0.0001) (Figure 4). Seventy iTTP patients without major depression (PHQ-9 score points < 10) had a median of 62 for the RS-11 score, which was comparable to the controls (median RS-11 score: 64, *p* = 0.65). The 32 iTTP patients with major depression (PHQ-9 score ≥10) had a median of 41.5 for the RS-11 score, which was significantly lower than that of the controls and the iTTP patients without major depression (*p* < 0.0001).

## 4. Discussion

For a long time, the survival of acute iTTP bouts was the main concern, but in recent years, the long-term consequences in survivors of iTTP have become more important. The prevalence of major depression in our iTTP patients was 21.6% and 34.5% for 2015 and 2016, respectively, far above the prevalence in our population controls (4.6%) and the reported 12-month prevalence in the German population (9.3%) [28]. These results are consistent with our previous findings [10] and with other studies showing a significantly increased point prevalence of depression from 19% up to 65% in iTTP survivors [6,8,9,29,30]. A strong association between chronic physical illness and depression has been reported [15,31]. Independent from the disease, the rate of 21.1% mood disorders in patients is significantly higher than in healthy individuals with 9.4% [31]. In addition, anxiety disorders have been documented, for example, in patients after a heart attack or stroke and with cancer [15,31]. Anxiety disorders in those with serious illnesses are just as common (22.9%) as depression (21.1%) [31]. Within the general population, anxiety disorders are the most common mental disorder, affecting about 15% [28]. Our examination of 87 iTTP patients revealed clinically relevant anxiety disorders in 19.5% and 17.6% for 2015 and 2016, respectively, as compared to a prevalence of 8.4% in the controls. Riva et al. found that 20% had anxiety disorders in their 35 TTP patients [7]. Regarding iTTP, survivors depression seems to be more common than anxiety disorders [7,32]. Gender, age and partnership [33] did not seem to be related to depression in our patients, whereas comorbidities did. This is congruent with the data of Härter et al. [31]. This is important since more than two-thirds of iTTP patients suffer from at least one other disease. Long-term data from the Oklahoma TTP registry showed a significantly higher prevalence of obesity, systemic lupus erythematosus, diabetes mellitus, arterial hypertension and major depression in survivors of iTTP [8,20,30]. Depression and anxiety are associated with increased morbidity and mortality. Martin-Subero et al. demonstrated for 803 inpatients over a follow-up period of 18 years that major depression was associated with a 2.4-times higher risk of mortality [34], independent of their disease. According to Cuijpers and Smit, mortality is increased regardless of the severity of depression [17]. Given that depressive symptoms affected up to 60% of our iTTP patients, together with low quality of life scores, antidepressive therapy seems mandatory. Lewis et al. [35], Cataland et al. [5] and Riva et al. [7] also reported a significantly compromised quality of life in iTTP patients. The number and severity of survived acute episodes do not seem to have a significant influence on the development and severity of depression [10]. An abnormal cerebral MRI scan during an acute episode does not implicate an increased likelihood of the development of depression or an anxiety disorder [32]. The survey on the attitude to life and resilience of our iTTP patients suggests that the patients were less resilient and optimistic, but nevertheless, not more pessimistic than the control group. The resilience of our iTTP patients was negatively related to the severity of their depressive symptoms. This is congruent with the findings, for example, in dry eye disease or cardiovascular disease [36,37]. According to other studies, more resilient individuals develop less depression and anxiety overall, regardless of whether they have a severe underlying disease [38,39,40,41]. The resilience may be reduced by the experience of a life-threatening disease and cognitive deficits, which further increases the risk of depression in iTTP.

### Limitations of the Study

Our study has limitations: First, we used self-report questionnaires. There was no examination by a clinician, such as in the studies by Han et al. [6]. However, we used questionnaires that have been widely validated in large cohorts of healthy subjects and patients. Second, only about 60% of our iTTP survivors participated in the self-evaluation study. Symptomatic patients may have been more motivated to answer the survey compared to asymptomatic patients. On the other hand, severely depressive patients may also have declined participation. The exact clinical data on the severity of the iTTP were not fully available for all patients, and comorbidities were not confirmed beyond the self-reporting. Finally, we do not have data on mental illness or resilience prior to the iTTP diagnosis.

## 5. Conclusions

The survivors of acute iTTP are significantly more likely to suffer from depressive and anxiety disorders as compared to the general population. The patients also reported a significantly compromised quality of life and perceived their cognitive performance as being significantly reduced. Overall, the iTTP patients were less optimistic and showed a significantly lower resilience, which in turn correlated strongly with the severity of the depression. It remains to be investigated whether psychological counseling in these long-term patients helps to improve neuropsychiatric disorders during long-term follow-ups. Furthermore, there is hope that new treatment strategies aiming at a fast resolution of the microvascular thrombotic process may improve long-term outcomes [42].

## Figures and Tables

**Table 1:** Characteristics of the responding and evaluable autoimmune thrombotic thrombocytopenic purpura (iTTP) patients in both surveys and of the healthy controls.

Heading	iTTP Patients		Healthy Controls
Time of survey	2015	2016	2016
Number (*n*)	89	84	134
Gender and age			
Female	69 (76%)	69 (82%)	108 (81%)
Male	20 (24%)	15 (18%)	26 (19%)
Age (years) median (min, IQR, max)	48 (18, 37–59, 86)	51 (21, 38–59, 87)	48 (19, 30–60, 79)
Data for age missing	6	7	0
Current partnership			
Yes	62 (73%)	60 (75%)	96 (74%)
No	23 (27%)	20 (25%)	34 (26%)
Data missing	4	4	4
Occupation, BMI, smoking status			
Employed	45 (51%)	41 (50%)	72 (55%)
Studying	3 (3%)	1 (1%)	14 (11%)
Retired	26 (30%)	22 (27%)	27 (21%)
Unemployed	2 (2%)	4 (5%)	2 (1%)
Working at home	4 (5%)	7 (8.5%)	5 (4%)
Other	8 (9%)	7 (8.5%)	10 (8%)
Data missing	1	2	4
BMI median (min, IQR, max)	26 (18, 23–31, 48)	28 (18, 24–32, 47)	24 (18, 21–26, 42)
Obesity (BMI ≥ 30)	24 (27%)	29 (35%)	12 (9%)
Data missing	0	2	4
Smoking	22 (25%)	21 (25%)	18 (14%)
Data missing	0	1	2
Physical activity			
Hardly active (1–2×/month)	25 (29%)	32 (39%)	22 (17%)
Quite active (3–4×/month)	10 (12%)	13 (16%)	24 (18%)
Active (1–2×/week)	33 (39%)	24 (30%)	45 (34%)
Very active (3–4×/week)	14 (16%)	8 (10%)	32 (24%)
Extremely active (>5×/week)	3 (4%)	4 (5%)	9 (7%)
Data missing	4	3	2
Number of comorbidities ^1^			
0	20 (22%)	10 (12%)	48 (36%)
1	25 (28%)	20 (24%)	43 (33%)
2	14 (16%)	23 (28%)	18 (14%)
≥3	30 (34%)	30 (36%)	23 (17%)
Data missing	0	1	2

^1^ Includes cardiovascular diseases, hypertension, gastrointestinal diseases, rheumatoid arthritis, diabetes mellitus, skin diseases, metabolic disorders, allergies, multiple sclerosis, chronic pulmonary diseases, chronic pain, thyroid diseases, obesity, cancer and other.

## Data Availability

The data presented in this study are available on request from the corresponding author. The data are not publicly available due to data privacy act.

## Supplementary Materials

The following are available online at https://www.mdpi.com/2077-0383/10/2/365/s1, Figure S1: Patient recruitment and response rates for the four surveys of the cohort of iTTP patients from Mainz, Table S1: Correlation of the PHQ-9 score (depressive symptoms) with sex, age, physical activity, partnership status and comorbidities in the 2015 and 2016 questionnaires.
