# Supplementary figures and images for: Influence of Personality, Resilience and Life Conditions on Depression and Anxiety in 104 Patients Having Survived Acute Autoimmune Thrombotic Thrombocytopenic Purpura
Source: J Clin Med. 2021 Jan 19;10(2):365. doi: 10.3390/jcm10020365 (PMC7835833; doi:10.3390/jcm10020365)

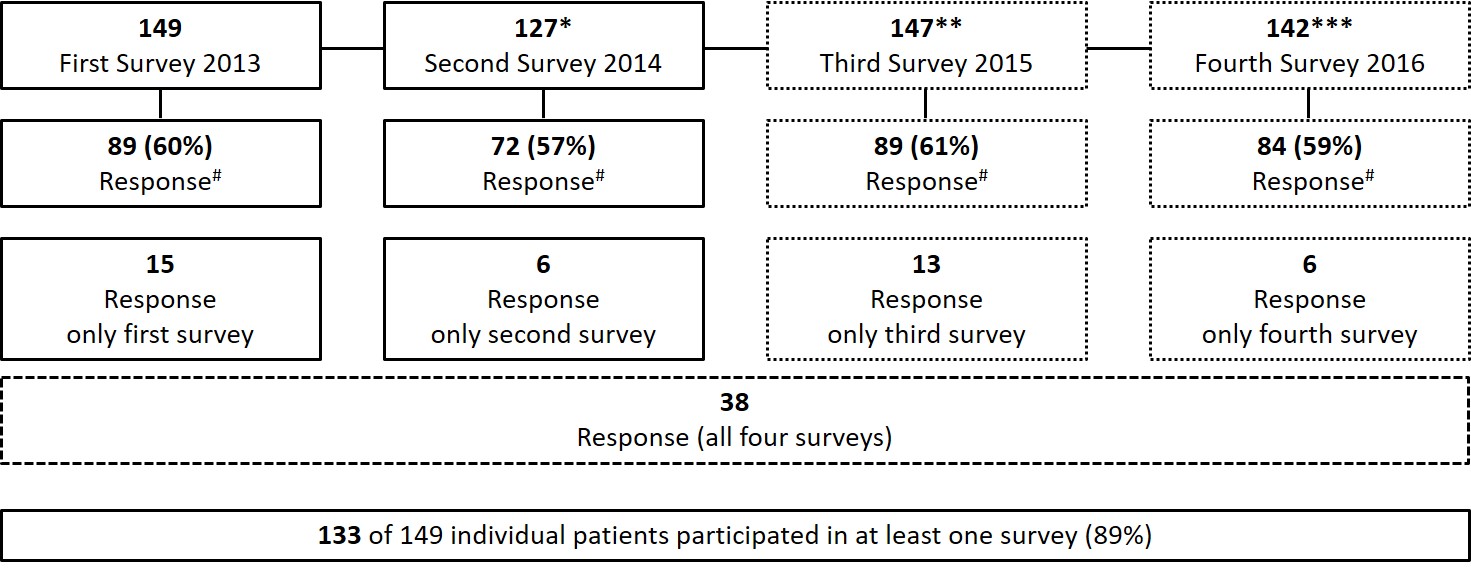

Supplement: Supplementary file 1 [file jcm-10-00365-s001.zip › Figure S1_Patient recruitment and response rates for the four surveys of the cohort of iTTP patients from Mainz.jpg]

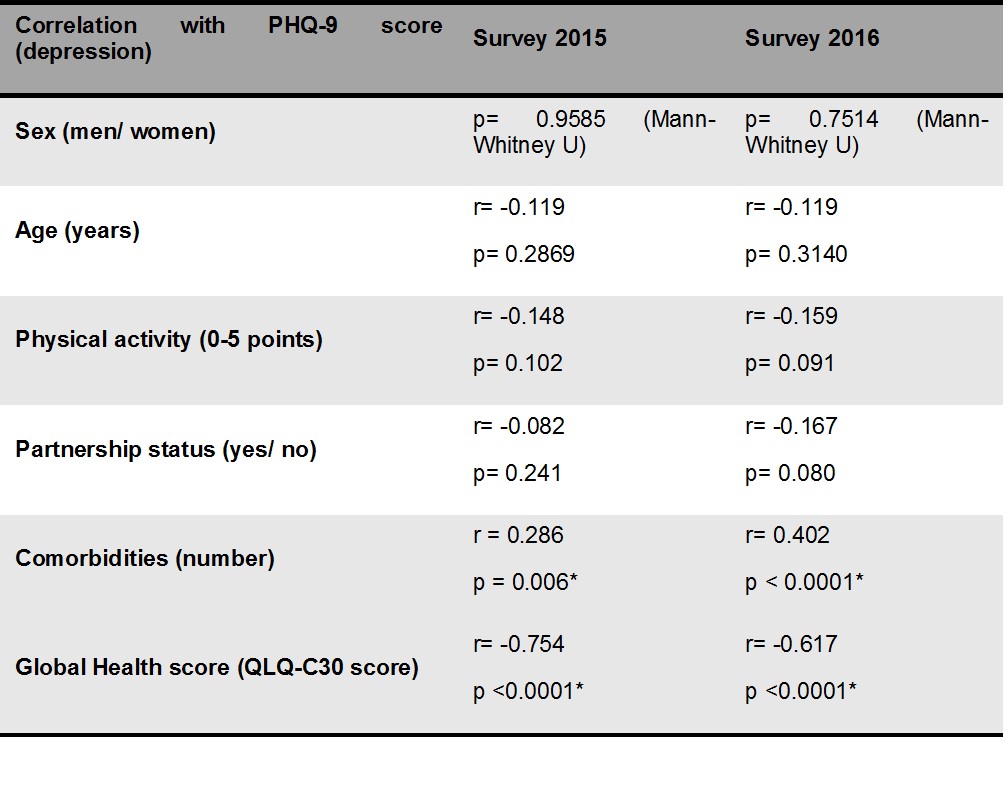

Supplement: Supplementary file 1 [file jcm-10-00365-s001.zip › Table S1_Correlation of the PHQ-9 score (depressive symptoms) with sex, age, physical activity, partner-ship status and comorbidities in the 2015 and 2016 questionnaires.jpg]
